# Clinical Notes

**Published:** 1888-04

**Authors:** H. W. Andrews


### CLINICAL NOTES.

EDITORS:—The following may be interesting to the worthy readers of your valuable journal. Miss A. came to the hospital as nurse in October. For some time before leaving home dreaded the new experience, and often freely expressing her feelings in tears. When she arrived had a severe headache, nausea, and later vomited a very bitter substance.

Being fair, gentle, and petite in form, Puls.$^{m}$ was given, which relieved the headache, but nausea and disgust at food continued for several days, with a painless, watery diarrhœa, which Ipec. and Arsen. in turn failed to subdue. Had noticed the *flushed face* and *trembling voice*, but attributed them to the surroundings, until one morning, as I entered the ward, a patient said, "Doctor, Miss A. *is so homesick she cries nearly all the time.*" Ah! is that the secret of all this suffering? I thought. Calling her to my room, I found her loneliness was almost beyond endurance; that letters from home were unopened for hours as the cheering contents only increased her suffering. This was her mental condition for over a week, which she was bravely trying to rise above. Caps. ann.$^{m}$, one dose, dry on the tongue, and before evening she was singing in a tone that indicated a contented mind. During her stay with us has not had a return of the mental condition, greatly to her delight as well as our own.

CASE II.—A gentleman said to me one day, "I have a peculiar sensation every time I walk, midway between groin and neck, *seems like a bunch of needles or pins sticking in deep; do not feel it when sitting.* Bry.$^{cm}$ subdued it within an hour.

H. W. ANDREWS.

