# Erratum

**Published:** 1888-05

**Authors:** 


ERRATUM.—In April issue, page 185, line 17th, for *neck* read *knee.*

